# (Healthy) Ageing: Focus on Iodothyronines

**DOI:** 10.3390/ijms140713873

**Published:** 2013-07-04

**Authors:** Pieter de Lange, Federica Cioffi, Elena Silvestri, Maria Moreno, Fernando Goglia, Antonia Lanni

**Affiliations:** 1 Dipartimento di Scienze e Tecnologie Ambientali, Biologiche e Farmaceutiche, Seconda Università degli Studi di Napoli, Via Vivaldi 43, Caserta 81100, Italy; E-Mail: pieter.delange@unina2.it; 2 Dipartimento di Scienze e Tecnologie, Università degli Studi del Sannio, Via Port’Arsa 11, Benevento 82100, Italy; E-Mails: federica.cioffi@unisannio.it (F.C.); silvestri@unisannio.it (E.S.); moreno@unisannio.it (M.M.); goglia@unisannio.it (F.G.)

**Keywords:** thyroid, ageing, thyroid hormone action, non-genomic pathways

## Abstract

The activity of the thyroid gland diminishes during ageing, but a certain tissue reserve of T3 and its metabolites is maintained. This reserve is thought to play a regulatory role in energy homeostasis during ageing. This review critically assesses this notion. T3 was thought to act predominantly through pathways that require transcriptional regulation by thyroid hormone receptors (TRs). However, in recent years, it has emerged that T3 and its metabolites can also act through non-genomic mechanisms, including cytosolic signaling. Interestingly, differences may exist in the non-genomic pathways utilized by thyroid hormone metabolites and T3. For instance, one particular thyroid hormone metabolite, namely 3,5-diiodo-l-thyronine (T2), increases the activity of the redox-sensitive protein deacetylase SIRT1, which has been associated with improvements in healthy ageing, whereas evidence exists that T3 may have the opposite effect. Findings suggesting that T3, T2, and their signaling pathways, such as those involving SIRT1 and AMP-activated protein kinase (AMPK), are associated with improvements in diet-induced obesity and insulin resistance emphasize the potential importance of the thyroid during ageing and in ageing-associated metabolic diseases.

## 1. The Thyroid Gland and Thyroid Hormones: A Brief Introduction

The thyroid gland produces 3,3′,5,5′-tetraiodo-l-thyronine (T4) (which accounts for over 90% of total thyroid hormone), 3,3′,5-triiodo-l-thyronine (T3), and 3,3′,5′-triiodo-l-thyronine (known as reverse T3, henceforth abbreviated as rT3). Upon release into the serum, the bulk of these hormones becomes bound to thyroxine binding globulin (TBG) and albumin, with only 0.03% of the total serum T4 and 0.3% of the total serum T3 being free and capable of entering tissue cells [1]. Most of the circulating T3 is actually produced in the periphery through the action of deiodinases, which catalyze the deiodination of the outer ring of T4, namely type I deiodinase (D1, present in liver and kidney) and type II deiodinase (present in brain, pituitary, and brown adipose tissue) [2,3]. There is evidence that the activity of D1 during the ageing process is influenced in an organ-specific manner [4] (see Section 2.2). 5-Deiodination of T4 by D1 and by type III deiodinase, which is present primarily in placenta, brain, and skin, produces rT3. Both rT3 and T3 are further deiodinated in the liver [5]. Local deiodinase activity thus delicately balances the tissue levels of thyroid hormone metabolites [6].

The cloning and characterization of thyroid hormone receptors (TRs) [7,8] led to the notion that T3 acts solely by genomic mechanisms, through its binding to TRs. Two major TR isoforms exist and are encoded on separate genes, designated as TRα and TRβ, and these reside on human chromosomes 17 and 3, respectively [9], which belong to the superfamily of nuclear hormone receptors, and they have distinct actions on metabolism, thus mediating the pleiotropic effects of T3 (for review, see [1]). The recognition of the tissue-specific expression of the TR isoforms (TRβ is expressed predominantly in liver, TRα in heart [10,11]) has had a potentially important outcome: namely, the development of TRβ-specific thyroid hormone analogs aimed at a lowering of serum cholesterol with minimal cardiac side-effects (which are known to be induced by T3) [12]. In recent years, thyroid hormone and its metabolites have been shown to have non-genomic effects too, via actions at the level of the cell membrane [13], the cytosol [14–17], the mitochondrion [18–20], or the nucleus [21,22]. Many aspects of the genomic and non-genomic actions of thyroid hormones have been reviewed elsewhere [1,12,13,23,24]. For clarity, the following sections of this review will deal only with the newly emerging thyroid hormone-related mechanisms involving factors related to the ageing process (such as sirtuin1 (SIRT1) and AMP-activated protein kinase (AMPK)). When discussing data relating to humans, this review will address only physiological changes in thyroid hormone levels during ageing, and will not refer to thyroid pathologies.

## 2. Thyroid Gland Activity: Ageing *vs.* Healthy Ageing

During ageing, the thyroid gland activity diminishes in humans, primates, and rodents [4,25–28]. Low thyroid function has been associated with increased longevity in humans [29–31], with a particularly strong case made in the Leiden Longevity study [31]. However, participants in that study were not pre-screened on health criteria or for demographic characteristics. Murine pituitary mutants (which reflect central hypothyroidism at the level of the pituitary) such as the long-lived Ames and Snell dwarf mice [32,33] display a similar longevity phenotype. Supplementation with T4 reduced the enhancement of life-span usually seen in Snell dwarf mice, but did not negatively influence the disease-resistance phenotype of those animals [34]. However, overt hypothyroidism after treatment for hyperthyroidism with radioiodine in the elderly causes death as does hyperthyroidism itself, and this has been shown to be prevented with T4 replacement therapy, which strongly suggests that the activity of the thyroid gland needs to be within a certain physiological range in order to assure longevity, at least in humans [35].

A possibility that should be considered is that in the elderly, age-related diseases, rather than ageing itself, are responsible for most morbidity. Population-based studies suggest that subtle changes in the activity of the thyroid gland may influence healthy ageing in euthyroid subjects. Indeed, subclinical hyperthyroidism has been associated with atrial fibrillation in the elderly [36]. Osteoporosis is one major contributor to morbidity and mortality in post-menopausal women [37]. Free T4 (FT4) levels that are high, but within the normal range, have been associated with increasing bone-loss and fracture-risk in both elderly males [38] and elderly females [39]. These effects have recently been found in mice to be associated with the action of TRβ [40]. A low level of FT4, within the normal range, could thus be a biomarker for decreased bone fracture-risk during ageing, but it is also an index for normocytic anaemia due to decreased erythropoiesis [41,42]. Indeed, in the elderly, a low FT4 is associated with a low erythrocyte index [41]. These studies may perhaps support the idea that reduced thyroid activity within the normal range during ageing can extend the length of life span, although not necessarily its quality. With this in mind, and on the basis of data derived from animal as well as human studies, the following sections will attempt to shed light on (a) how changes in thyroid hormone metabolism in various tissues might contribute to the maintenance of energy homeostasis during ageing; and (b) how certain thyroid hormone metabolites might positively influence healthy ageing.

### 2.1. Selective Age-Related Decline in Thyroid Hormone Levels, Evidence from Animal Studies

Ageing in male rats is characterized by a decreased secretion of thyroid hormones, despite an unchanged plasma TSH, a situation suggestive of impairments in the hypothalamo-pituitary-thyroid axis [43]. Silvestri *et al*. [4] reported that, compared to those in young (6-month-old) male rats, serum concentrations of TT4 at 12 and 24 months were lower, by 31 and 53%, respectively, and those of FT4 were lower by 48% and 79%, respectively. By contrast, TT3 and FT3 levels were lower only in the 24-month-old rats (by 71% and 50%, respectively). Rhesus monkeys show a similar picture: that is, TT3 levels tend to show a decrease, albeit not a significant one, only at a pronounced age (>20 years), whereas FT4 and TT4 levels decline significantly (by approximately 35%) over the adult life-span. Serum TSH also decreases significantly with age in the monkey (by more than 50%), with no gender differences [28].

The finding of a reduced life-span in the usually long-lived Snell dwarf mouse upon T4 supplementation [34] points towards a direct inverse relationship between thyroid hormone levels and longevity. It may, however, be that exogenous hormone administration delivers an excess of T4 to organs that would not normally receive T4 during ageing, which could reduce longevity. Instead, higher local levels of T3 and/or its metabolites in organs that maintain their need for T3 (or its metabolites) as ageing progresses could be beneficial. Indeed, as described in the next section, there is evidence for a tight organ-specific control of T3 levels during ageing [4].

### 2.2. Toward an Understanding of the Age-Related Control of Peripheral T3 Levels

The reduced D1 activity in the thyroid and liver of 26-month-old male rats (*vs*. 4-month-old male rats) is indicative of an impaired conversion of thyroid hormones in peripheral tissues with age [43]. By contrast, in the pituitary of aged rats, there is an increase in the D1 activity, suggesting an adaptive mechanism to compensate for the low circulating levels of thyroid hormones which leads to an unchanged concentration of T3 within the pituitary and consequently to unaltered plasma levels of TSH in aged rats [43]. However, information is sparse concerning intra-organ thyroid hormone levels during ageing. The first report on the responses of different organs to ageing-related thyroid hypofunction was published by Silvestri and colleagues [4]. They studied the contribution of liver and kidney to thyroid hormone homeostasis during ageing in the rat. Both liver and kidney take up and conserve major quantities of T4 and T3, and they each exhibit high rates of thyroid hormone metabolism and excretion. Both organs depend strongly on T3; indeed, this hormone controls the expression of around one-tenth of the hepatic genes [44] and it is crucial for growth, hemodynamics, and osmoregulation in the kidney [45]. Cellular uptake of T3 occurs through the action of plasma membrane transporters, such as the thyroid hormone-specific and active monocarboxylate transporter 8 (MCT8) [46]. Modulation of tissue-specific expression of MCT8 during ageing would thus be expected to lead to fluctuations in intracellular T4 and T3 levels. Interestingly, Silvestri *et al*. [4] have shown that in old male rats (aged 24 months), MCT8 levels are reduced in the liver, but not in the kidney. A second way of altering intracellular T3 levels during ageing, namely, by modulating organ-specific D1 activity, has also been addressed by the same group. In rat liver, D1 activity declines progressively with age, but in kidney the decline is slower, and only significant in old age (24 months). Nuclear TRβ protein levels decrease in both of those organs with age, whereas nuclear TRα1 levels are unaltered. Notably, the same authors showed that total cellular TRβ protein levels increase during ageing in both liver and kidney (in the latter, TRα protein also increases), which might suggest that certain extra-nuclear non-genomic actions, several of which being reported to depend on TRs [13,14,22,24] are more pronounced during ageing. However, to our knowledge no information exists in the literature on this subject. The above data show that, during ageing, the primary role in T3 production shifts from the liver to the kidney. The resulting maintenance of T3 levels may suggest that during ageing certain cellular processes are in constant need of T3 or its metabolites, at least in the rat.

### 2.3. Thyroid Hormone Metabolism in Humans, and Maintenance of Type I Deiodinase Activity during Ageing

Most clinical studies show a clear decline in serum FT3 levels with age [25,26], whereas serum rT3 increases [25,26] and FT4 levels remain unchanged [25,26,47–51]. In addition, the serum levels of the thyroid hormone metabolites 3,3′-diiodo-l-thyronine, 3′,5′-diiodo-l-thyronine, and 3,5-diiodo-l-thyronine (henceforth abbreviated as T2) decrease with age in humans [52]. Serum TSH levels were previously considered to be reduced in the elderly [25,26] but this may have been due to incorrect sampling, and to the fact that circulating TSH has different forms (ranging from different grades of glycosylation to sialylation) and bioactivities, which needs to be considered when data on immunoreactive TSH are interpreted. Indeed, recent evidence shows that TSH concentrations increase with healthy ageing, despite unchanged circulating FT4 levels [47–51], albeit that this is also associated with an increased prevalence of metabolic syndrome-related diseases in the elderly [53,54]. An overview of the serum thyroid hormone levels in relation to ageing is given in Table 1. As described above, animal studies have shown that local deiodinase activity levels are maintained during ageing, which underlines their importance in the physiology of ageing [4]. Human studies seem to support this. The deiodinases have been shown to be selenoproteins [55,56]. Modest selenium deficiency is common worldwide and is associated with age-related diseases such as cancer, heart disease, and immune dysfunction [57]. In modest selenium deficiency, the actitivies of essential selenoproteins are maintained, whereas those of proteins classified as non-essential are lost, with the exception of the thyroid D1, which is currently classified as conditionally essential [57]. Maintenance of the activities of selenoproteins such as the peripheral deiodinases depends on adequate selenium uptake, which may thereby contribute to the prevention of disease in later life.

## 3. Healthy Ageing: Key Factors Involved, and Their Relationship with the Thyroid and Iodothyronines

### 3.1. Central Factors in Metabolic Control: Sirtuins and AMPK

Sirtuins are NAD^+^-dependent deacetylases of which there are seven in humans and other mammals, termed SIRT1-7 [58]. Since reports were published indicating that sirtuins extend life-span in yeast [59], roundworms [60], and fruit flies [61], these proteins have attracted considerable attention, and research on the effects of sirtuins on life-span has been extended to studies on mammals. SIRT1 levels decline with age in tissues in which mitotic activity decreases over time, but not in tissues with constant mitotic activity [62]. It has been suggested that SIRT1 may promote genomic stability in mammalian cells since it represses repetitive DNA [63]. Indeed, in mice, loss of SIRT1 induces transcriptional changes similar to those occurring in the ageing brain, while overexpression of SIRT1 can suppress such changes [63]. In addition, genetic overexpression of SIRT1 in mice results in a lower level of DNA damage, decreased expression of the ageing-associated gene p16^Ink4a^, improved general health, and a reduced incidence of spontaneous carcinomas and sarcomas, but has no effect on life-span *per se* [64]. Genetic loss of another sirtuin, SIRT6, in mice causes severe metabolic effects, rapid ageing, and death at four weeks of age [65]. SIRT3, a mitochondrial sirtuin, has been shown to control cardiac dilatation during ageing, and through its direct control of cardiac mitochondrial fatty acid oxidation, it has been suggested to counteract the pressure-overload-mediated decompensation and maladaptive phenotype of the ageing heart [66]. Nutrient or caloric restriction causes major metabolic changes that increase longevity throughout the animal kingdom [67,68]. The metabolic effects of the sirtuins in fruit flies [61] and mammals [67] have been shown to be reminiscent of those of caloric restriction, and indeed it is now known that caloric restriction exerts its beneficial effects during ageing via SIRT1 activation [67]. In line with this, it has been shown that polymorphisms in the SIRT1 gene are related to an increased body mass index and increased risk of obesity [69]. In a recent study, a polymorphism in the human SIRT1 gene has been associated with reduced mortality risk and increased glucose tolerance [70]. Because of the observations that sirtuins have beneficial effects on health, and that they positively affect age-related diseases (for a recent extensive review, see [71]), pharmaceutical agents targeting sirtuins are currently being developed [72].

Similarly, AMPK is required for the control of metabolic pathways serving to maintain energy homeostasis under conditions of metabolic stress, such as fasting and caloric restriction, during which the cellular AMP/ATP ratio is increased [73,74]. Like the sirtuins, AMPK has been shown to extend life-span in the roundworm [75]. One group of targets of AMPK is proteins belonging to the Forkhead transcription factor (FOXO) family [76]. In the roundworm, the AMPK-FOXO pathway has been shown to be crucial for increased life-span under conditions of caloric restriction [76], but as yet no information on this issue exists in mammals. Given its ability to modulate mitochondrial activity, mammalian AMPK is also a strong candidate for an agent controlling ageing processes. Qiang *et al*. [77] found a decrease in AMPK activity with age in rat skeletal muscle. In contrast, Reznick *et al*. [78], did not observe age-related changes in AMPK activity in mouse skeletal muscle but they did find that stimulation of AMPK activity by AICAR and exercise occurs in young, but not in old, mice. Such stimulation was accompanied by an increase in mitochondrial density in young mice, but not in old ones, thus suggesting that impaired AMPK activation is linked to reduced mitochondrial biogenesis during ageing [78]. It is unclear whether or not these results can be generalized to tissues other than skeletal muscle. Indeed, one study [79] showed increased basal AMPK activity in the liver in 24-month-old mice (*vs.* five-month-old mice), although the same study also found that the responsiveness of AMPK activity declines with age. Thus, more research is needed before we can fully define the role of AMPK in ageing, especially in the light of the beneficial effects that AMPK seems to have on pathways associated with ageing and age-related disease [80].

### 3.2. Resveratrol: Providing a Link among Ageing, AMPK, SIRT1, and Iodothyronine Function

Resveratrol (RSV), a plant secondary metabolite found in red grapes, has been associated with extended life-span in yeast, fruit flies, and roundworms [81,82]. The beneficial effects of resveratrol on metabolism and in age-retardation partially mimic those of caloric restriction [83,84]. The discovery that RSV is an activator of both SIRT1 [85,86] and AMPK [85,87,88] has currently stimulated a search in many laboratories for natural compounds or endogenous hormones that might act through similar pathways to improve metabolic parameters, including thyroid hormone metabolites (see Section 3.4 and further). Interestingly, RSV increases serum T3 levels in ovariectomized rats, without inducing morphological changes in the thyroid [89], and RSV stimulates TSH-independent iodide uptake and elevates the levels of the sodium-iodide symporter (NIS), each by around three-fold, in a rat thyroid cell-line FRTL-5 [90]. As the RSV target, SIRT1, has been shown to increase pituitary TSH secretion through deacetylation and activation of phosphatidylinositol-4-phosphate 5 kinase (PIP5K)γ [91], these data may suggest that there exists an association between the alleviating effect of RSV on the metabolic discomforts of ageing, SIRT1 action, and increased thyroid function.

### 3.3. Oxidative Stress and Ageing

Ageing is accompanied by an imbalance between an increase in the production of reactive oxygen species (ROS), and antioxidant defenses. Skeletal muscle function regresses with ageing: complementary DNA microarray analysis of rat skeletal muscle has revealed an overall decrease in the expression of genes, including those involved in energy metabolism and mitochondrial respiration. The only genes displaying increased expression were (a) those involved in the transition from glycolytic to oxidative fibers and (b) presumably as a compensatory measure, those involved in the defense against ROS production. The proteomic profile changed accordingly [92]. One protein involved in the defense against ROS is uncoupling protein 3 (UCP3). Evidence supporting the idea that the age-related reduction in UCP3 levels is associated with a rise in ROS production was published by Nabben *et al*. [93], who showed that the increase in mitochondrial ROS generation associated with ageing is blunted in UCP3 over-expressing mice. In rat muscle, UCP3 translocates lipid hydroperoxide and mediates lipid hydroperoxide-dependent mitochondrial uncoupling [94], thus protecting the mitochondrial matrix from this very aggressive molecule while exerting a thermogenic role.

It is known that disturbances in redox homeostasis lead to ischemia-reperfusion injury [95]. T3 prevents reperfusion injury by enhancing ROS production, thus triggering the redox upregulation of cytoprotective proteins affording preconditioning against ischemia-reperfusion injury in liver [96], heart [97], and kidney [98]. In the liver, this protective action of T3 has been associated with increased phosphorylation of AMPK, resulting in increased activity of this protein. Age represents a threat for myocardial infarction, since ischemic preconditioning becomes less effective with age [99], an effect that, to judge from the evidence above, may be associated with the age-related reduction in serum T3.

### 3.4. Glycemic Control and Ageing

The increasing prevalence of obesity worldwide and the consequent rise in type 2 diabetes mellitus is expected to result in mortality increasing by 50% within the next 10 years [100]. Numerous studies have shown that impaired insulin signaling contributes to reduced longevity through increased ROS formation and increased pro-inflammatory signals, and is associated with telomere shortening (reviewed in [100]). The relationship between the thyroid and insulin is complex (for review, see [101] and references therein). Historically, hyperthyroidism has been associated with insulin resistance, and more recently hypothyroidism has also been linked with decreased insulin sensitivity. Delicate balances exist between the effect of thyroid hormones on liver lipogenesis, adipose lipolysis, and general lipid and glucose homeostasis. Whether the effects of thyroid hormones on insulin signaling are agonistic or antagonistic depends on the interplay between different organs, and these vary under different nutritional conditions [101]. Population-based clinical studies indicate that polymorphisms in genes that take part in thyroid hormone-regulated metabolic pathways, such as the TSH receptor [102] and D1 [103], alter their functions and influence both body composition and metabolic diseases, such as insulin resistance [103].

Both AMPK and SIRT1 are major players in glycemic control. In metabolically active mammalian tissues, depending on the experimental settings, AMPK may regulate the activity of SIRT1 [104], or *vice versa* [85,105]. Moreover, AMPK and SIRT1 signaling pathways converge: these metabolic key enzymes each activate crucial factors involved in cellular energy metabolism. Peroxisome proliferator γ-activated receptor coactivator (PGC)-1α is activated both by AMPK (through phosphorylation [106]), and by SIRT1 (through deacetylation [86,107]). The FOXO family of proteins represent a second mutual activation target, being phosphorylated by AMPK [108] and deacetylated by SIRT1 [109]. Upon phosphorylation [108] and/or deacetylation [110], both PGC-1α and members of the FOXO family increase lipid catabolism as well as both mitochondrial synthesis and respiration, key features of caloric restriction. Since it is evident that AMPK and SIRT1 signaling pathways intertwine, it is perhaps not surprising that under certain conditions these two pathways are activated simultaneously.

Recent evidence indicates that AMPK is involved in the glycemic control exerted by thyroid hormones. Notably, T3 administration in animal models rapidly phosphorylates AMPK at Thr^172^ in muscle [16,17], resulting in increased PGC-1α expression [16], increased carnitine palmitoyl transferase activity (providing increased uptake of fatty acids into the mitochondria for oxidation), and increased fatty acid oxidation [17]. Further, T3 increases both glucose uptake and glycolysis through activation of the Akt/PKB pathway [14,17], by enhancing glucose transporter (GLUT)1 expression and phosphofructokinase (PFK) activity [14,17] and by increasing GLUT4 membrane levels [17]. It should be stressed that in absence of adequate inhibition of central and peripheral deiodinase activities, the effects derived from individual thyroid hormone metabolites cannot properly be dissected. Injection of T3 into euthyroid rats results in T2 accumulation in serum and liver within 24 hours, coinciding with a peak in metabolic rate [111]. Thus, a possibility that cannot entirely be excluded is that part of the presumed T3-mediated effects in studies, in which deiodinase activity is not inhibited, [14,95] are not due to T3 itself.

**Figure 1:**
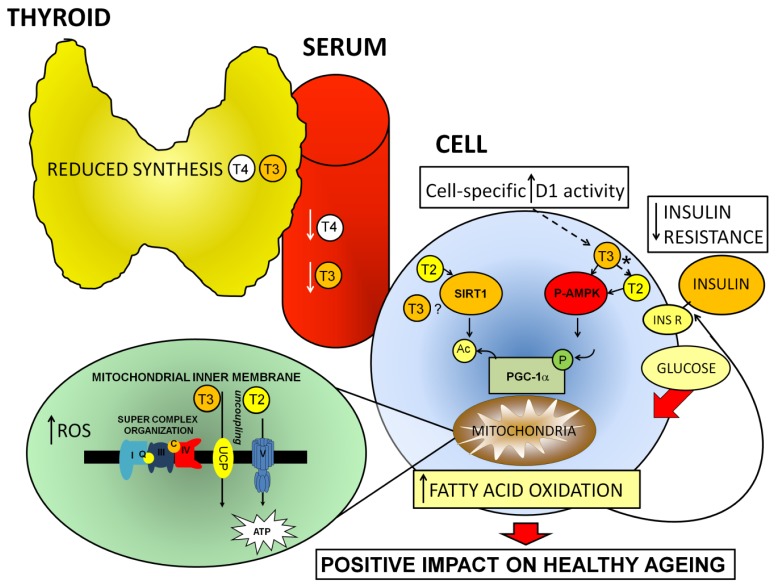
Ageing-dependent decline in thyroid activity, leading to compensatory increases in cellular D1 activity and thyroid hormone metabolites that may, through non-genomic pathways, be involved in amelioration of ageing related metabolic disturbances. For abbreviations: see text. * The conversion of T3 into T2 occurs via a D1 independent, as yet unidentified pathway.

In rodent models, high-fat diets (HFD) are given to create a model of diet-induced obesity, resulting in systemic insulin resistance [21,77,112–115]. Hepatic overexpression [112] and specific activation of SIRT1 [113] ameliorates HFD-induced insulin resistance. In addition, the thyroid hormone metabolite T2 prevents insulin resistance through SIRT1 activation in HFD-fed rats [21]. Conversely, inactivation of SIRT1 results in an insulin resistant and obese phenotype in HFD-fed mice [114]. The idea of a role for SIRT1 in ameliorating insulin resistance, and thus increasing life-span, which came originally from animal studies, was recently confirmed in a human study in which reduced mortality risk and increased glucose tolerance are associated with polymorphisms in the human SIRT1 gene [70]. One consequence of hyperglycemia in humans is an increased incidence of nephropathy [116], and it has recently been shown that polymorphisms within SIRT1 are associated with susceptibility to diabetic nephropathy [117]. Actually, activation of SIRT1 by T2 ameliorates diabetic nephropathy in rats [118], and SIRT1-activation by the specific agonist SRT1720 in mice and by T2 in rats has been shown to result in indirect activation of AMPK in the longer term [21,113]. So far, results from animal studies on the prevention of insulin resistance by AMPK-inactivation seem contradictory. Indeed, mice deprived of the regulatory β2 subunit of AMPK have been reported to be hyperglycemic, glucose intolerant, and insulin resistant when maintained on a HFD [74]. Inactivation of the catalytic α1 subunit, paradoxically, has been shown not to promote insulin resistance in HFD-fed mice [115]. Interestingly, the thyroid hormone metabolite T2 activates AMPK in rat skeletal muscle, an effect associated with increased fatty acid oxidation and uncoupled mitochondrial respiration [18]. T2 has also been shown to be a potent activator of hepatic lipid metabolism in a HFD rat model, leading to normalization of body weight and adipose mass, a process associated with activation of AMPK in liver [15]. Intriguingly, T2 has recently been discovered to be a direct activator of SIRT1 [21]. The initial effects of T2 on liver lipid oxidation have been shown to be SIRT1-dependent, with longer-term effects being associated with increased SIRT1 as well as with increased AMPK activity [21]. Through SIRT1-dependent deacetylation of PGC-1α and sterol receptor element binding protein (SREBP)1c, T2 upregulates the expression of genes involved in fatty acid oxidation, and downregulates that of genes involved in lipogenesis, respectively. The authors have observed that T2 has no effect on TRβ-dependent transcription, but one cannot entirely exclude the involvements of TRs in the T2-mediated effects on hepatic SIRT1 activity *in vivo*, even though *in vitro* assays carried out in the same study showed that T2, like RSV, directly activated SIRT1 in the absence of TRs [21]. For a schematic overview of how increases in local D1 activity despite diminished thyroid activity might positively affect the ageing process, via local production of T3 and T2 (albeit that the T3–T2 conversion is not mediated by D1 itself [3]) and activation of AMPK and SIRT1, see Figure 1.

### 3.5. Evidence for Contrasting Effects of T3 and T2 on SIRT1 Activity

Evidence exists that T3, in direct contrast to T2, inhibits SIRT1 *in vitro* [21]. A recent study addressed the effect of T3 on SIRT1 activity *in vivo* [22]. Transgenic mice harboring a dominant-negative mutation in TRβ, created to serve as a model of thyroid hormone resistance [119], have higher hepatic SIRT1 activity, and so do hypothyroid wild-type mice, compared to their respective wild-type littermates [22]. Conversely, in hypothyroid mice, T4 supplementation reduces liver SIRT activity [22]. In addition, fasted mice (having undetectable serum T4 levels and a 26% lower T3 level) display increased SIRT1 activity, which becomes normalized to control levels after T4 supplementation [22]. These data point toward an inverse relation between T3 levels and SIRT1 activity. A seemingly contrasting result came from a study that provided strong evidence that modulation of hepatic gene transcription in hypothyroid rats by T3 is partially dependent on the presence of SIRT1 [120], but the authors did not show *in vivo* induction of SIRT1 activity or deacetylation of SIRT1 targets, such as PGC-1α or SREBP-1c. Therefore, current evidence suggests that T2 and T3 have opposite effects on SIRT1 activation, pointing toward a specific beneficial role for T2 during ageing. However, further research is needed to test this notion.

## 4. Mitochondrial Organization during Ageing: Proteomic and Transgenic Approaches

Proteome analysis of skeletal muscle in the ageing rat has revealed that although mitochondrial activity is reduced (reflected by a reduction of ATP synthase activity), mitochondrial inner membrane respiratory complexes are organized in supercomplexes with a modified structural organization, allowing the mitochondria to respire more efficiently [92]. Similar results have been obtained in rat cerebral cortex [121], but in the rat heart supercomplex formation has been reported to decline with age [122]. The altered supercomplex organization in rat skeletal muscle mitochondria results in a more coupled respiration [92]. Uncoupling, though, may also protect against the increased threat caused by ROS during ageing, as illustrated by the defensive role against hydroperoxide accumulation exerted by UCP3 [94]. Actually, uncoupled respiration *per se* does not seem to reduce life span, but rather to increase it. Indeed, in three different transgenic mouse strains that specifically over-express the uncoupling protein 1 gene in skeletal muscle leading to increased uncoupling of mitochondrial respiration, there were delays in death and in age-related disease [123], while in skeletal muscle isolated from long-lived mice, an increase in mitochondrial uncoupling has been observed [124]. In addition, modest respiratory uncoupling has been shown to preserve mitochondrial function in ageing human muscles [125]. Seemingly in contrast, in skeletal muscle, an age-dependent decline in the mitochondrial UCP3 protein content has been observed. Kerner *et al.* [126] reported that UCP3 protein was significantly less abundant in skeletal muscle mitochondria from 24-month-old rats than in those from eight-month-old rats. This decline is associated with an inhibition of uncoupled respiration [92,126]. Nevertheless, the observations that increased uncoupling may be beneficial for the ageing process [123–125], and the findings (a) that T3 induces UCP3 levels [127,128] (with a peak reached at 65 hours after injection), resulting in increases in skeletal muscle mitochondrial uncoupling and resting metabolic rate in hypothyroid rats [128], and (b) that the thyroid hormone metabolite T2 induces uncoupling (within one hour after injection) in skeletal muscle of hypothyroid rats as well [18] may point toward a beneficial role for thyroid hormone metabolites in the process of ageing. This role for thyroid hormone metabolites may especially apply to T2, which, in contrast to T3 has no deleterious cardiac effects in rats [12,15].

## 5. Conclusions

From the current, accumulated evidence relating to the role of iodothyronines in ageing, it could be deduced that local maintenance of appropriate levels of thyroid hormone, or its metabolites, may be important for the maintenance of metabolic events that are key to the prevention of age-related disease. The current idea that thyroid hormones may have non-genomic effects that can contribute positively to healthy ageing is intriguing, and deserves further attention. In particular, SIRT1- and AMPK-activation by local levels of thyroid hormone metabolites in physiological settings could contribute to an improved prospect for health during ageing. In that respect, seeking ways of manipulating the local levels of active thyroid hormone metabolites could be an interesting and potentially rewarding avenue of exploration.

## Figures and Tables

**Table 1:** Selective decline of thyroid hormone serum levels in various species during ageing. Percentages of the initial values are given. Abbreviations: N.D. = not determined, N.S. = not significant.

Hormone	Young	Adult	Old	Species and References
TSH	100	100	150	Human [47–51]
	100	100	50	Rhesus monkey [28]
	100	100	100	Rat [4]

T4	100	100	100	Human [25,26]
	100	100	65	Rhesus monkey [28]
	100	69	47	Rat [4]

FT4	100	100	100	Human [25,26,47–51]
	100	100	65	Rhesus monkey [28]
	100	52	21	Rat [4]

T3	100	100	50	Human [25,26]
	100	100	<100 (N.S.)	Rhesus monkey [28]
	100	100	29	Rat [4]

FT3	100	100	50	Human [25,26]
	100	100	50	Rat [4]

3,3′-T2	100	N.D.	62	Human [52]

3,5′-T2	100	N.D.	42	Human [52]

3,5-T2 (T2)	100	N.D.	53	Human [52]
